# Comprehensive Modeling in Predicting Biodiesel Density Using Gaussian Process Regression Approach

**DOI:** 10.1155/2021/6069010

**Published:** 2021-06-17

**Authors:** Bingxian Wang, Issam Alruyemi

**Affiliations:** ^1^School of Mathematics and Statistics, Huaiyin Normal University, Huaian, Jiangsu 223300, China; ^2^Fouman Faculty of Engineering, College of Engineering, University of Tehran, Fouman, Iran

## Abstract

In this study, four Gaussian process regression (GPR) approaches by various kernel functions have been proposed for the estimation of biodiesel density as the functions of pressure, temperature, molecular weight, and the normal melting point of fatty acid esters. Comparing the actual values with GPR outputs shows that these approaches have good accuracy, but the performance of the rational quadratic GPR model is better than others. In this GPR model, RMSE = 0.47, MSE = 0.22, MRE = 0.04, *R*^2^ = 1, and STD is equal to 0.3. In addition, for the first time, this study shows that the effective parameters affect the biodiesel density. According to this analysis, it was shown that among the input parameters, pressure has the greatest effect on the target values with a relevancy factor of 0.59. This study can be used as a suitable and valuable work/tool for chemical and petroleum engineers who attempt environment protection and recovery improvement.

## 1. Introduction

Recently, in various countries, the issue of energy is deep and complex [1–3]. The greenhouse gas concentration in the atmosphere has been increased because the consumption of fossil fuels increased, and in that case, the earth's temperature increased too [4, 5]. For the solution to these problems, several agreements have been signed to reduce greenhouse gas emissions [6]. There are so many reasons but the main reasons for blooming the renewable energy resources are the aim of controlling the emission of pollutants and the implementation and credibility of these agreements [7, 8]. The two things that mainly can form biodiesel are the oils that come from vegetables and the fat of an animal's body [9]. Biodiesel is a combination of fatty acid alkyl esters [10]. Ethanol and methanol are some of the alcohols that the oils that come from vegetables and the fat of animal's body are transesterification catalytically by them [10]. Biodiesel is a clean fuel to burn, and it is not a toxic fuel because of low concentrations of sulfur. In that case, this fuel has the minimum bad effect on the emission of greenhouse gas changes, and also unlike fossil fuels, the biodiesel fuel has a less negative effect on our environment too [11, 12]. Additionally, in diesel engines, we can use biodiesel fuels lonely or use this fuel with fossil diesel because it improves engine life [13], although this fuel costs more than petroleum-based diesel and has higher viscosity too. Some other disadvantages of this fuel are lower oxidation stability, higher cloud point, and lower energy content in comparison with petroleum-based diesel [14]. The observational and modeling studies for the establishment of these properties become important because the concentration on usages of the biofuels and their properties have been grown [15, 16]. For example, the density of biodiesel is the important property that has a significant matter in the thermophysical process. One of the important topics in diesel fuels is the investigation of density because diesel fuels have so many technical and economic parts for the usage of the fuel and also the environmental effects [17–19].

For estimating the properties of fossil fuels, lots of investigations have been carried out in the literature and also there are different advancements in this study, such as a new way for guessing many properties of fuels consisting of surface tension and viscosity has been proposed by Queimada and his colleagues [20]. Barati-Harooni and his colleagues estimate interfacial tension between oil and brine with developed least-squares support vector machine in terms of pressure, salinity, and temperature [21]. Rostami and his colleagues correlate interfacial tension of hydrocarbon and water using genetic programming (GP) with an *R*-squared of 0.910 [22]. The Kay's model is a model that can determine the density of biodiesels that was estimated by Pratas and his colleagues [17]. The alkane density with an average absolute relative deviation of 60% was predicted by Gahk and his colleagues [23]. Miraboutalebi with the help of his coworkers estimate cetane numbers with a root mean squared error (RMSE) of 2.530 and *R*-squared of 0.950 by implementing an artificial neural network (ANN) [24]. On the other hand, the cetane number in terms of fatty acid methyl ester (FAME) was predicted by Mostafaei with developing the adaptive neuro-fuzzy inference system [25].

For biodiesel properties, there are some investigational searches in the literature. For example, the density of biodiesels was measured by Paratas et al. at atmospheric pressure with ten individual samples in temperatures between 278.150 and 373.170 K [17]. The viscosities and densities of three individual mixtures of methylcyclohexane and fatty acid methyl esters were obtained by Li and his colleagues in the atmosphere in temperatures between 293.150 and 324.150 K [26]. The density of soybean oil biodiesel was experimentally determined by Aitbelale and his colleagues at temperatures between 298.150 and 393.150 K and pressures up to 140.0 MPa [27]. The surface tension values were measured by Aitbelale et al. for three different biodiesels in temperature and pressure of 473.0 K and 7.0 MPa [27].

The lack of sufficient accuracy and difficulties of computations cause more attention in the aforementioned literature. On the other hand, much attention has been paid to artificial intelligence methods to a precise solution in order to model different processes [28–33]. One of the things that are crucial for the process design and the same operation is the accuracy of thermophysical properties. The development of a precise and low-cost advancement towards the estimation of biodiesel properties is worthy because of the necessity of these properties most importantly in the clean energy resource topics. For this job, the density of biodiesel fuels has been researched thoroughly. The development of the GPR algorithm model is the main purpose of this research for the estimation of biodiesel density. This algorithm is better for the estimation of individual properties compared to other models because of the independence of this algorithm from the outliers. In the process of development of this model, the reliability and accuracy of the collected dataset are important so for the first time for the identification of suspected data points of biodiesel density, a throughout analysis has been carried out. On the other hand, the effects of input variables on the output have been researched statistically as an important part of this work.

## 2. Material and Methods

### 2.1. Gaussian Process Regression

In recent years, the trend of neural networks, which is a branch of artificial intelligence, has made significant progress in solving problems related to the engineering field. The main disadvantage of this method is overfitting, which of course can be improved by adjusting the weight [34, 35].

Of course, setting these parameters is also complex and difficult, and to solve this problem, a conventional mathematical method called Bayesian network is used. It should be noted that this method is probabilistic and uses Bayesian interference to calculate the probability [36]. This network plots each variable graphically, and these variables are connected by an arc, and each variable shows its knowledge content as a distribution of probabilities. It should be noted that the potential specificity of BNs is of great importance for assessing uncertainty. High distribution leads to more uncertainty. One of the factors influencing the increase of complex prior distribution on functions in the Bayesian method to neural networks is the prior overweight [37].

Gaussian process regression is a developed method for the abovementioned problem. It should be noted that this method is nonparametric. The advantages of the GPR algorithm include measuring uncertainty for predictions and working well on small data. It is worth noting that the GPR method has important advantages over Bayesian, including simplicity, nonlinearity, easy generalization, and has several dimensions. The model parameters are determined using the sample training information in the GPR method.

The GP model is obtained by linking previous knowledge to the current process of modeling and integrating real laboratory data.

One of the salient and important differences between the old methods of machine learning and GPR is not finding the best approximation with experimental points and is a complete BN core. GPR works periodically by obtaining posterior distributions on models. In the following, we will explain how to create GP regression [38].

Randomly selected points *T* = {*x*_*L*,*i*_, *y*_*L*,*i*_} and *L* = {*x*_*T*,*i*_, *y*_*T*,*i*_}, *i* = 1, 2, 3, ⋯, *n*, which are test and learning data from a particular distribution, are assumed as follows [39]:

(1) $$\begin{array}{c} T=\left\{x_{L,i},y_{L,i}\right\},L=\left\{x_{T,i},y_{T,i}\right\},i=1,2,\cdots ,n. \end{array}$$

As an important point, we remind that the model parameters are adjusted based on the learning data [40].

As the input and goal data, respectively, *x* and *y* have been assumed that noise has affected them.

The general formula of the GPR described as follows [41]:

(2) $$\begin{array}{c} y_{L,i}=f\left(x_{L,i}\right)+e_{L,i},n=1,2,3,\cdots ,n. \end{array}$$

In the abovementioned equation, *X*_*L*_ and *Y*_*L*_ denote independent and objective variables of the training data, and subsequently, the *ε*^~^*N*(0, *σ*_noise_^2^*l*_*n*_) denotes for the observation noise with the independent Gaussian distribution that *I*_noise_ symbolizes and *σ*_noise_^2^ the variance of noise and unit array [42].

*Note*: GP assumes the output *f*(*x*) is random. So the following equation is obtained:

(3) $$\begin{array}{c} y_{T,i}=f\left(x_{T,i}\right)+\varepsilon_{T,i},i=1,2,3,\cdots ,n. \end{array}$$

In the above equation, *y*_*T*_ and *x*_*T*_ represent the goals and independent variables and the *f*(*x*) represents a Gaussian process with covariance function *k*(*x*, *x*′) and mean function *m*(*x*).

(4) $$\begin{array}{c} f\left(x_{L,i}\right)\sim \text{GP}\left(m\left(x\right),k\left(x.x^{\prime }\right)\right). \end{array}$$

But in practice, the exact determination of *m*(*x*) can be complicated, so the value of *m*(*x*) is taken to be zero to make the calculations easier, so we have the following [43]:

(5) $$\begin{array}{c} f\left(x_{L,i}\right)\sim \text{GP}\left(0,k\left(x.x^{\prime }\right)\right). \end{array}$$

From Eqs. (2) and (5), we can conclude the following equation:

(6) $$\begin{array}{c} y\sim N\left(0,k\left(x.x^{\prime }\right)+\sigma_{\text{noise}}^{2}I_{n}\right). \end{array}$$

A better representation of the variables mentioned in the text above can be provided as follows [44]:

(7) $$\begin{array}{c} \left[\begin{array}{c} \overset{⟶}{f_{L}}\\ \overset{⟶}{f_{T}} \end{array}\right]\sim N\left(0,\left[\begin{array}{cc} k\left(x_{L}.x_{L}\right) & k\left(x_{L}.x_{T}\right)\\ k\left(x_{T}.x_{L}\right) & k\left(x_{T}.x_{T}\right) \end{array}\right]\right), \end{array}$$

(8) $$\begin{array}{c} \left[\begin{array}{c} \overset{⟶}{{ɛ}_{L}}\\ \overset{⟶}{{ɛ}_{T}} \end{array}\right]\sim N\left(0,\left[\begin{array}{cc} \sigma_{\text{noise}}^{2}I_{n} & 0\\ 0 & \sigma_{\text{noise}}^{2}I_{n} \end{array}\right]\right). \end{array}$$

The following Gaussian function is obtained by adding the sum of Eqs. (7) and (8):

(9) $$\begin{array}{c} \left[\begin{array}{c} \overset{⟶}{y_{L}}\\ \overset{⟶}{y_{T}} \end{array}\right]\sim N\left(0,\left[\begin{array}{cc} k\left(x_{L}.x_{L}\right)+\sigma_{\text{noise}}^{2}I_{n} & k\left(x_{L}.x_{T}\right)\\ k\left(x_{T}.x_{L}\right) & k\left(x_{T}.x_{T}\right)+\sigma_{\text{noise}}^{2}I_{n} \end{array}\right]\right). \end{array}$$

Therefore, the previous distribution of *Y*_*T*_ is obtained from Gaussian conditions as below:

(10) $$\begin{array}{c} \left(\overset{⟶}{y_{T}}\;|\;\overset{⟶}{y_{L}}\right)\sim N\left(\mu_{T},\Sigma_{T}+\sigma_{\text{noise}}^{2}I_{n}\right). \end{array}$$

And values *Σ*_*T*_ and *μ*_*T*_ are assumed as follows:

(11) $$\begin{array}{c} \Sigma_{T}=k\left(x_{T}.x_{T}\right)=k\left(x_{T}.x_{T}\right)+\sigma_{\text{noise}}^{2}I_{n}-k\left(x_{T}.x_{L}\right).\left(k{\left(x_{L}.x_{L}+\sigma_{\text{noise}}^{2}I_{n}\right)}^{-1}\right.k\left(x_{L}.x_{T}\right),\\ \mu_{T}=m\left(\overset{⟶}{y_{T}}\right)=k\left(x_{T}.x_{L}\right).\left(k{\left(x_{L}.x_{L}+\sigma_{\text{noise}}^{2}I_{n}\right)}^{-1}\right.\overset{⟶}{y_{T}}. \end{array}$$

In GPR modeling, the following theoretical concept is obtained by predicting the output of experimental data through independent variables and training data. From the above equations, it can be concluded that the covariance and the mean function of both together with the Gaussian distribution represent a GP.

To better predict the goals of the developed GPR model, the selection of the core function in the training phase is of great importance. Therefore, in this research, to find the best kernel function, we use four different and conventional kernel functions, of course, with changes.

These functions are described as follows [45]:

- Rational quadratic covariance function

(12) $$\begin{array}{c} k_{\text{RQ}}\left(x.x^{\prime }\right)=\sigma^{2}{\left(1+\frac{x-x^{\prime 2}}{2al^{2}}\right)}^{-a}. \end{array}$$

In rational quadratic covariance function equation *σ*^2^, 1, *a* > 0 represents the variance, length, and weight scale changes.

(ii) Squared exponential covariance function

(13) $$\begin{array}{c} k_{\text{SE}}\left(x.x^{\prime }\right)=\sigma^{2}\left(-\frac{x-x^{\prime 2}}{l^{2}}\right). \end{array}$$

(iii) Exponential covariance function

(14) $$\begin{array}{c} k_{E}\left(x.x^{\prime }\right)=\sigma^{2}\exp\left(-\frac{x-x^{\prime }}{l}\right). \end{array}$$

(iv) Matern covariance function

(15) $$\begin{array}{c} k_{M}\left(x.x^{\prime }\right)=\sigma^{2}\frac{2^{1-v}}{\Gamma \left(v\right)}{\left(\sqrt{2v}\frac{x-x^{\prime }}{l}\right)}^{v}K_{v}\left(\sqrt{2v}\frac{x-x^{\prime }}{l}\right). \end{array}$$

The symbol represents the gamma function and *x*, *y* are positive parameters, and the adjusted *K*_*v*_ is the Bessel function [46].

In the Matern equation, exponential covariance and quadratic are important functions. The Matern function is an exponential when the value of *V* = 0.5 and also when the exponential is a square when *V* is inclined to infinity. Since the Matern equation has a greater degree of freedom, it also performs better than the other two [47].

Since the GPR method is nonparametric, the learning stage tries a lot to modify the parameters of the above equations.

### 2.2. Data Collection

The dataset containing 2117 real density points has been collected from different resources. References to this data have been reported elsewhere [48]. These points are in the pressure range of 0.1-129.78 MPa, melting point of 238.15-304.15 K, molecular weight of 186.291-310.514 g/mol, and temperature range of 278.36-413.15 K. The density values are different between 769.4 and 951.3 kg/m^3^ in terms of these conditions.

## 3. Results and Discussion

As mentioned before, here, four various GPR algorithms including kernel functions in Matern, rational quadratic, exponential, and square exponential forms are used for estimating the density of biodiesels. To evaluate the precision of these algorithms, statistical analysis of parameters is determined as follows:

(16) $$\begin{array}{c} \text{MRE}=\frac{1}{n}\overset{n}{\underset{i=1}{\sum }}\;\frac{\left|y_{\exp.,i}\right.-\left.y_{\text{pred}.,i}\right|}{y_{\text{pred}.,i}},\\ \text{MSE}=\frac{1}{n}\overset{n}{\underset{i=1}{\sum }}{\left(y_{\exp.i}-y_{\text{pred}.i}\right)}^{2},\\ \text{RMSE}=\sqrt{\text{MSE}}=\sqrt{\frac{1}{n}\overset{n}{\underset{i=1}{\sum }}{\left(y_{\exp.i}-y_{\text{pred}.i}\right)}^{2},}\\ \text{STD}=\sqrt{\frac{1}{n-1}\overset{n}{\underset{i=1}{\sum }}{\left(\frac{y_{\exp.i}-y_{\text{pred}.i}}{y_{\exp.i}}\right)}^{2},}\\ R^{2}=1-\frac{\sum_{i=1}^{n}{\left(y_{\text{pred}.i}-y_{\exp.i}\right)}^{2}}{\sum_{i=1}^{n}{\left(y_{\text{pred}.i}-\bar{\;y_{\exp.i}}\right)}^{2}}. \end{array}$$

As you see in Table 1, *R*^2^ values of rational quadratic, Matern, exponential, and square exponential forms are equal to 1. According to other statistical parameters, the rational quadratic form depicts a better performance than the other kernel functions. In this kernel function form, RMSE, MSE, MRE, and STD are obtained 0.47, 0.22, 0.04, and 0.30, respectively. These values exhibit the rational quadratic formability in the forecast of density values.

Comparing the results of *R*^2^ from this table with the results published by Abooali et al. who used SGB and GP models to predict biodiesel density, it was concluded that the models presented by us have a higher ability to predict the target values because the *R*^2^ values for these two models were obtained 0.99988 and 0.99635, respectively [48].

**Figure 1:**
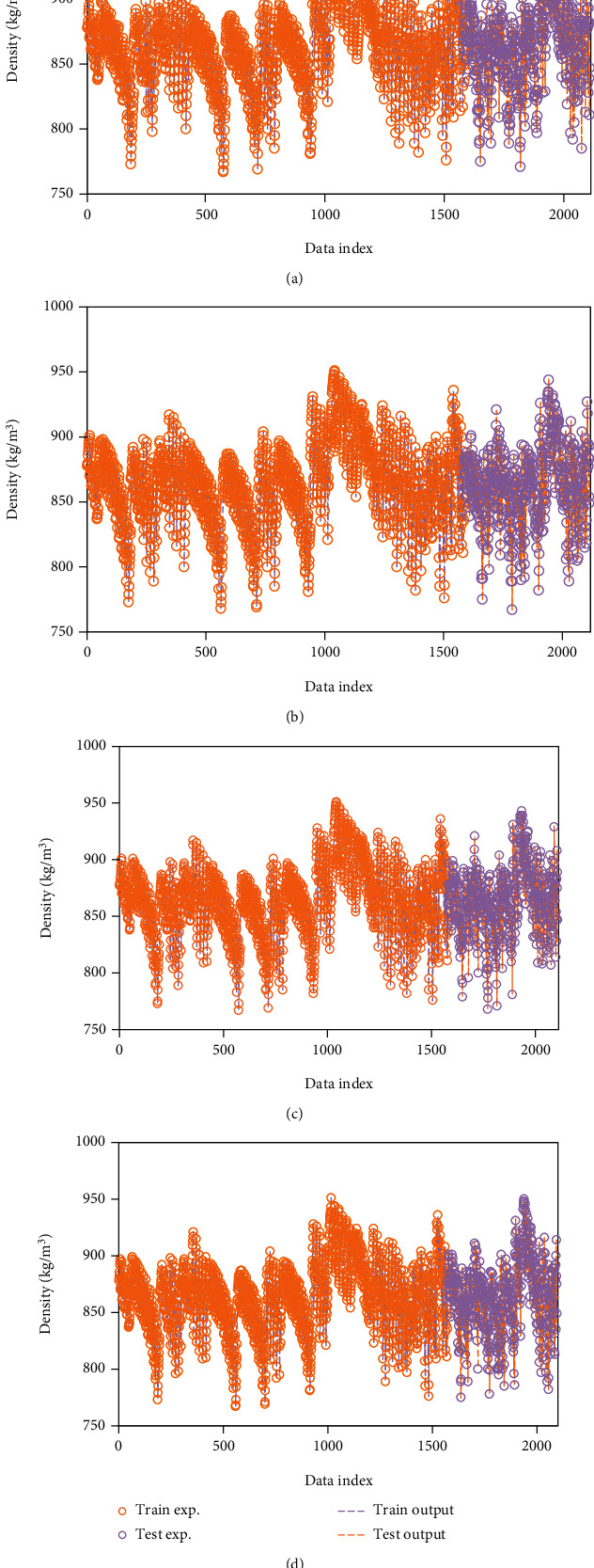
The performance of the values predicted against their corresponding actual values using different kernel functions including (a) exponential, (b) Matern, (c) squared exponential, and (d) rational quadratic.

To make a better decision about all these models, Figure 1 shows the experimental and estimated density values, simultaneously. In this figure, there is a good agreement between the real density values and GPR outputs.

**Figure 2:**
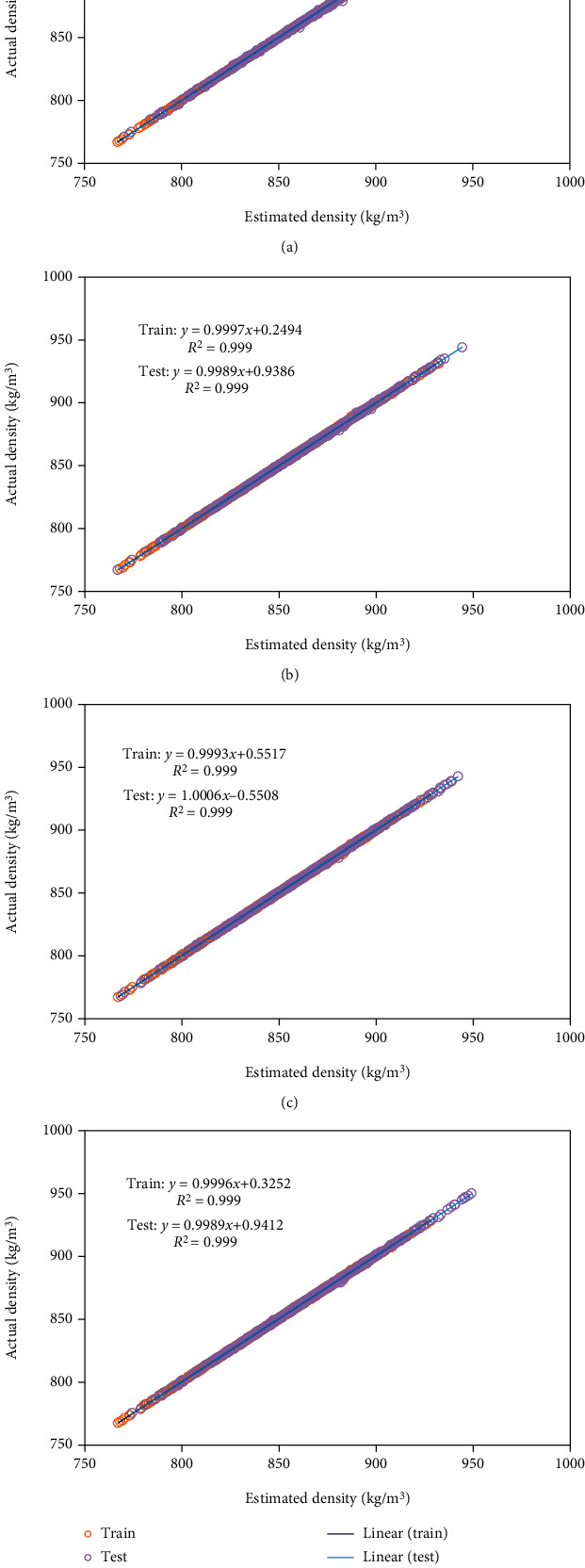
Cross plots of predicted output values for different kernel functions including (a) exponential, (b) Matern, (c) squared exponential, and (d) rational quadratic.

Also, the cross/regression plot of predicted and real density has been shown in Figure 2.

**Figure 3:**
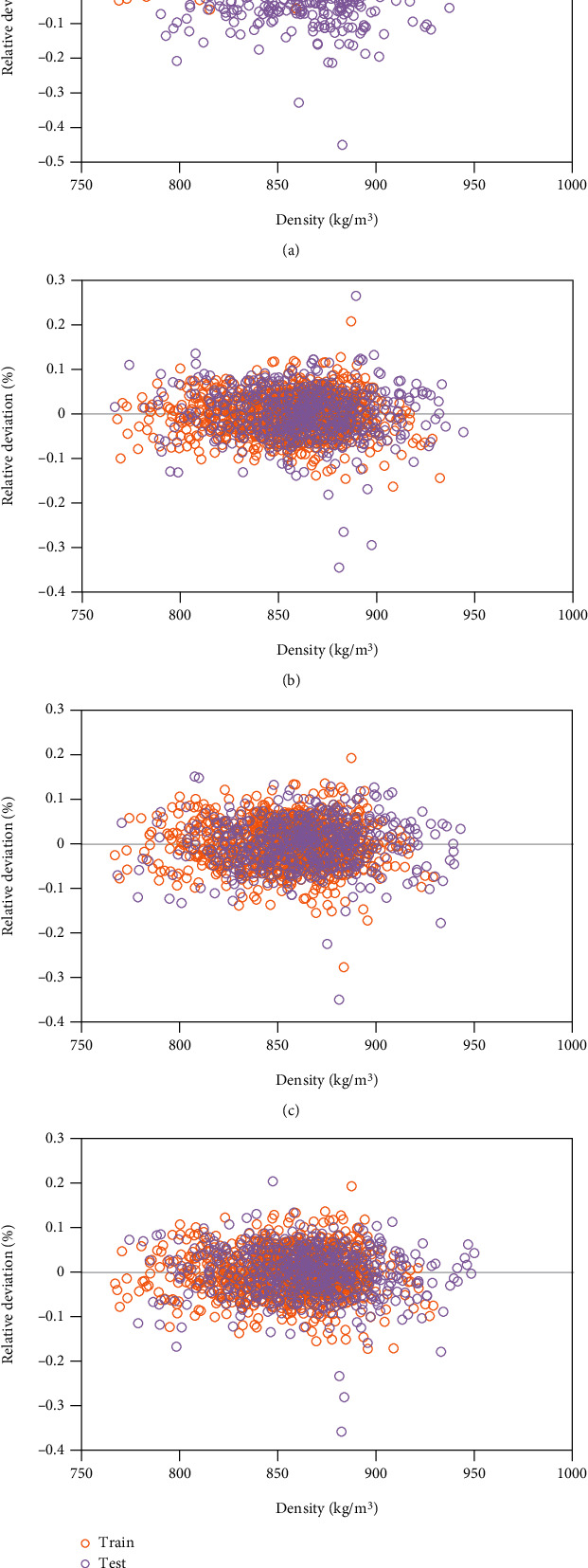
The relative deviation between the real density and GPR outputs using different kernel functions including (a) exponential, (b) Matern, (c) squared exponential, and (d) rational quadratic.

To express the quality of GPR outputs, we refer to the density data located on bisector lines in the analysis. Moreover, Figure 3 shows the relative deviation between the real density and GPR outputs.

The accuracy of these density point data affects the validity of models. In this examination, too many data points have been used. It is important to know that these data may have errors due to measurements done in laboratories. So, these types of data are separated from the other data points. In this regard, some strongly developed strategies are required to remove these data and enhance the model accuracy. Here, the separation of these suspected data is accomplished by the Leverage method. In this method, after the determination of residual values, a Hat matrix is created as follows [49]:

(17) $$\begin{array}{c} H=A{\left(A^{T}A\right)}^{-1}A^{T}, \end{array}$$

where *A* is an *i* × *j* dimensional matrix and *i* and *j* are defined for the model parameters and training points, respectively. Then, the critical leverage limit is computed by *i* and *j* as follows:

(18) $$\begin{array}{c} H^{*}=\frac{3\left(b+1\right)}{a}. \end{array}$$

**Figure 4:**
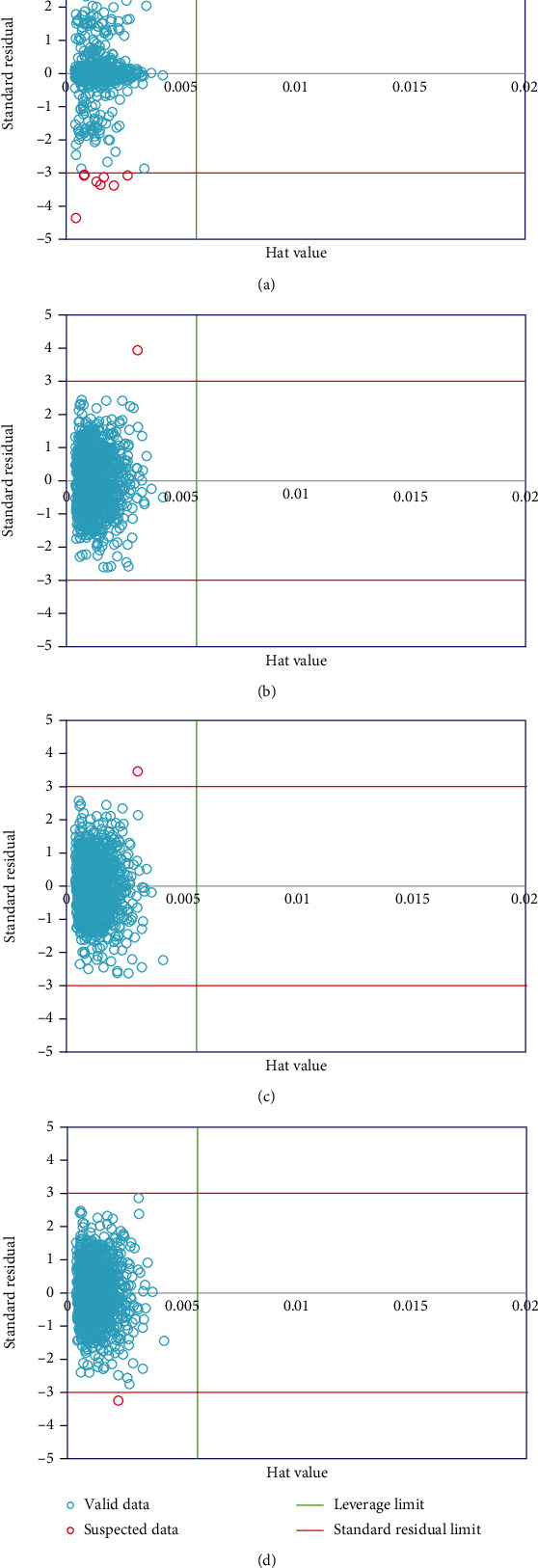
Determination of suspected data point for the various kernel functions including (a) exponential, (b) Matern, (c) squared exponential, and (d) rational quadratic.

According to William's plot, as shown in Figure 4, we can separate the suspected data obtained as residuals from Hat values. Here, the leverage limits in the green line and two red lines are considered as residuals. So, the data points located outside these lines are considered as suspected data. As you see, for exponential, Matern, squared exponential, and rational quadratic forms, 14, 1, 1, and 1 points are considered as the suspected data, respectively, among 2217 points.

**Figure 5:**
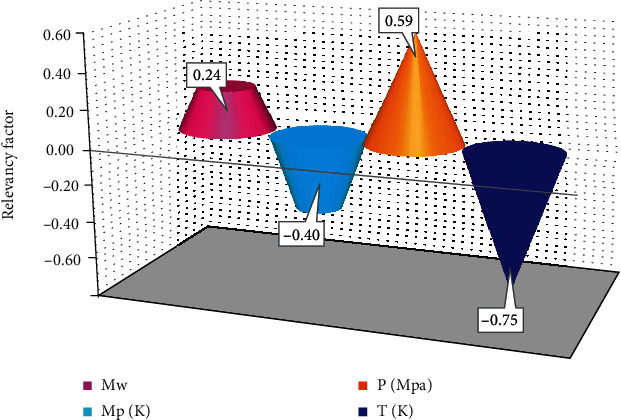
Sensitivity analysis on various input parameters.

In this study, the suggested GPR algorithms create a relationship between density and inputs. So, sensitivity analysis is utilized to show this relationship affects the output. In this regard, to determine the most efficient variable for density, the relevancy factor, *r*, in the range of -1 and 1, is used. If the absolute value of *r* is large, it can affect the density further. The less and more relation to density is shown by negative and positive *r* values, respectively. The *r* is calculated as follows [50]:

(19) $$\begin{array}{c} r=\frac{\sum_{i=1}^{n}\left(x_{K,i}-{\bar{x}}_{k}\right)\left(Y_{i}-\bar{Y}\right)}{\sqrt{\sum_{i=1}^{n}{\left(x_{K,i}-{\bar{x}}_{k}\right)}^{2}\sum_{i=1}^{n}{\left(Y_{i}-\bar{Y}\right)}^{2}}}, \end{array}$$

where *X*_*k*.*i*_ and *Y*_*i*_ are inputs and outputs, respectively. The $\bar{X_{k}}$ and $\bar{Y}$ are the average of inputs and that of outputs, respectively.  Figure 5 shows that the higher temperature and melting point lead to less density. Also, the temperature is considered the most efficient parameter for density. In addition, parameters such as pressure and molecular weight have a direct relationship with this target.

## 4. Conclusion

In this work, four various kernel functions including Matern, rational quadratic, exponential, and square exponential functions have been used for GPR algorithms to compute the density of biodiesels. To prepare and validate these algorithms, a large database containing 2217 actual data is gathered. It is concluded that the proposed models have highly precise to predict real data. The rational quadratic GPR model has shown greater performance compared with other models. In this model, the calculations show that RMSE = 0.47, MSE = 0.22, MRE = 0.04, *R*^2^ = 1, and STD is equal to 0.3. Other analyses also confirmed the accuracy of this model, which indicates that this attractive and simple model can be used in biodiesel-related industries.

## Figures and Tables

**Table 1:** Obtained statistical parameters to evaluate the performance of the models.

Model	Phase	*R* ^2^	MRE (%)	MSE	RMSE	STD
GPR (exponential)	Train	1.000	0.01	0.01	0.11	0.10
	Test	1.000	0.05	0.38	0.61	0.45
	Total	1.000	0.02	0.10	0.61	0.29

GPR (Matern)	Train	1.000	0.04	0.18	0.42	0.27
	Test	1.000	0.04	0.25	0.50	0.33
	Total	1.000	0.04	0.19	0.50	0.28

GPR (squared exponential)	Train	1.000	0.04	0.21	0.46	0.30
	Test	1.000	0.04	0.23	0.48	0.32
	Total	1.000	0.04	0.22	0.48	0.30

GPR (rational quadratic)	Train	1.000	0.04	0.22	0.47	0.30
	Test	1.000	0.04	0.22	0.47	0.30
	Total	1.000	0.04	0.22	0.47	0.30

## Data Availability

The data are stated in the article.
